# Non-local competition drives both rapid divergence and prolonged stasis in a model of speciation in populations with degenerate resource consumption

**DOI:** 10.1186/1742-4682-9-56

**Published:** 2012-12-27

**Authors:** Nicholas Atamas, Michael S Atamas, Faina Atamas, Sergei P Atamas

**Affiliations:** 1 Epic Games, Cary, NC 27518, USA; 2 Harvard Law School, Cambridge, MA 02138, USA; 3 BioBitField, Ellicott City, MD 21042, USA; 4 University of Maryland School of Medicine, Baltimore, MD 21201, USA

**Keywords:** Evolutionary mechanisms, Speciation, Adaptation

## Abstract

The theory of speciation is dominated by adaptationist thinking, with less attention to mechanisms that do not affect species adaptation. Degeneracy – the imperfect specificity of interactions between diverse elements of biological systems and their environments – is key to the adaptability of populations. A mathematical model was explored in which population and resource were distributed one-dimensionally according to trait value. Resource consumption was degenerate – neither strictly location-specific nor location-independent. As a result, the competition for resources among the elements of the population was non-local. Two modeling approaches, a modified differential-integral Verhulstian equation and a cellular automata model, showed similar results: narrower degeneracy led to divergent dynamics with suppression of intermediate forms, whereas broader degeneracy led to suppression of diversifying forms, resulting in population stasis with increasing phenotypic homogeneity. Such behaviors did not increase overall adaptation because they continued after the model populations achieved maximal resource consumption rates, suggesting that degeneracy-driven distributed competition for resources rather than selective pressure toward more efficient resource exploitation was the driving force. The solutions were stable in the presence of limited environmental stochastic variability or heritable phenotypic variability. A conclusion was made that both dynamic diversification and static homogeneity of populations may be outcomes of the same process – distributed competition for resource not affecting the overall adaptation – with the difference between them defined by the spread of trait degeneracy in a given environment. Thus, biological degeneracy is a driving force of both speciation and stasis in biology, which, by themselves, are not necessarily adaptive in nature.

## Introduction

### Evolutionary stasis: continuous elimination of outlying forms

Although selection-driven heritable phenotypic divergence is the key mechanism of adaptive evolution, prolonged evolutionary stasis is common in populations remaining in stable or near-stable environments [1-8]. Evolutionary stasis of populations is commonly associated with phenotypic trait homogeneity, or the relative rarity of outlying trait values [1-8]. Such populations converge towards the most adaptive trait values, allowing for maximal exploitation of the available resources. Stasis is likely the predominant mode of evolution [9], but the mechanisms of stasis are not well understood. Stabilizing selection is widely assumed to provide the best explanation of trait homogeneity in static populations, but the direct evidence for stabilizing selection in the wild is far from overwhelming and many stable traits persist even in widely varying environmental conditions [9]. Moreover, many species remain remarkably static over long time periods, but then undergo rapid phenotypic evolution and genetic differentiation, often only to transition to the next state of stasis [8,10-17]. Evoking stabilizing selection alone to explain stasis is insufficient, because it is difficult to reconcile the frequency with which evolutionary stasis is observed with the well-known abundance of genetic variation in traits, which ensures the capacity for evolvability [9].

In addition to stabilizing selection, the theory of canalization suggests that “the constancy of the wild-type must be taken as evidence of the buffering of the genotype against minor variations not only in the environment in which the animals developed but also in its genetic make-up” [18]. In other words, mechanisms of stability against random genetic and short-term environmental perturbations – ensuring robustness, or canalization [19] – must have evolved, to “protect” the efficiency of resource exploitation by a phenotypically homogenous population against non-directional stochastic variations in environments and genotypes. A specific example of this phenomenon is Hsp90-mediated genetic capacitance [20,21]. Normally, Hsp90, an ubiquitous molecular chaperone, assists in the folding of diverse proteins, particularly signal transducers. When its function is impaired, numerous previously silent mutations manifest themselves phenotypically [20-25]. Thus, Hsp90 serves as an evolutionary “capacitor” that silences phenotypic manifestation of cryptic genetic variation, thus creating conditions for accumulation of cryptic mutations. Under stress, this silencing is broken, and the previously accumulated cryptic genetic variation becomes manifest, offering a rich substrate for natural selection. Thus, Hsp90 has evolved as a promoter of evolvability by making genetic variations cryptic or manifest, depending on the environmental conditions.

Both stabilizing selection and trait canalization are adaptive mechanisms. However, alternative explanations are also possible. For example, canalization may be a result of constraints that are imposed by the developmental process, which are controlled by a network of interacting transcriptional regulators [19]. In this view, canalization is an inevitable consequence of the complexity of the developmental process and not a mechanism that has evolved to buffer the effects of minor environmental fluctuations on phenotypes [19]. In this work, we propose that a separate mechanism not affecting overall adaptation contributes to the evolutionary stasis of relatively homogenous populations of heritable phenotypes as well as to divergent speciation: non-local competition for resource stemming from phenotypic degeneracy. Before discussing degeneracy, it is necessary to consider an important commonality between diversification and homogenous stasis of traits.

### Suppression of intermediate and diversifying forms: a common phenomenon in evolutionary divergence and stasis

Although prolonged stasis and rapid differentiation are diametrically opposed in their effects on population diversity (the former narrows and the latter broadens the heterogeneity of phenotypic characters), the underlying mechanisms of these two processes are not necessarily conflicting. Moreover, there is a possibility that common mechanisms contribute to these opposing processes, which must be viewed together as periods of “punctuated stasis” rather than independent evolutionary processes. One important mechanistic commonality in prolonged stasis and rapid diversification is the apparent suppression of certain trait values.

In the evolutionary divergence of new species, intermediate, or transitional, forms are suppressed and, ultimately, excluded. Darwin reflected “… that species come to be tolerably well-defined objects, and do not at any one period present an inextricable chaos of varying and intermediate links …” [26]. However, the process of diversification into species appears counter-intuitive, as Darwin has pointed out: “As according to the theory of natural selection an interminable number of intermediate forms must have existed, linking together all the species in each group by gradations as fine as are our existing varieties, it may be asked, Why do we not see these linking forms all around us? Why are not all organic beings blended together in an inextricable chaos?” [26]. It will not, perhaps, be an overstatement to say that many evolutionary biologists will readily echo Darwin’s confession, “This difficulty for a long time quite confounded me” [26]. Numerous mechanisms of speciation have been suggested, significantly expanding the spectrum of such mechanisms initially proposed by Darwin, and the interest in this topic continues to grow exponentially (see Figure 1 in [27]). Genetic mutability, geographic and reproductive isolation, polyploidization, genetic drift, hybridization, gene flow – all appear to go hand-in-hand with natural and sexual selection processes leading to speciation. Despite this spectrum of mechanisms, it can be argued that none of them specifically addresses the issue of the rarity of intermediate forms.

**Figure 1:**
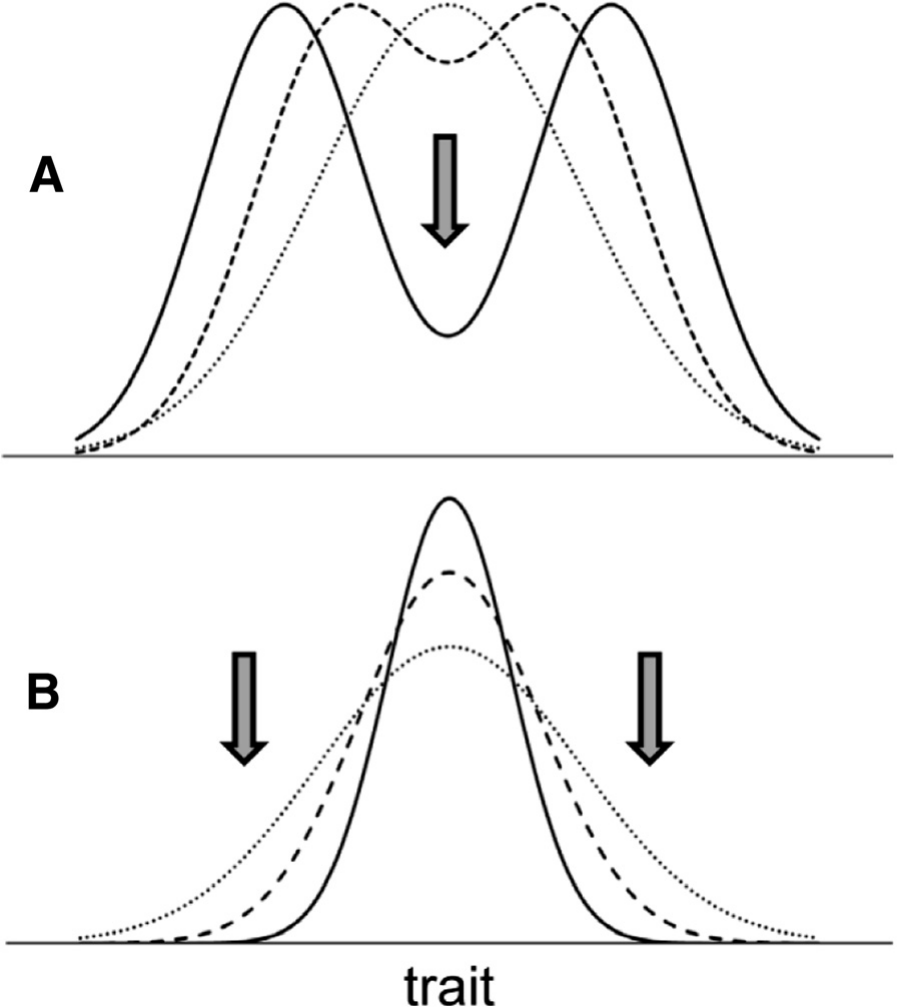
**Schematic depiction of dynamics in diversifying (A) and static (B) populations.** In each panel, the initial distribution of a defining trait is shown with a dotted line. The population transitions to subsequent distributions are indicated with dashed and then solid lines. In **A**, further dynamics lead to a complete separation of the newly forming populations. In **B**, trait homogeneity may increase further. In either diversification or stasis, suppression of certain forms occurs: the intermediate forms in **A** and the diversifying forms in **B** become suppressed and may ultimately be excluded at trait values indicated with arrows.

A similar concern applies to static populations. As discussed above, static populations converge toward trait homogeneity. As a result, outlying trait values – analogous to transitional forms in divergent evolution – are rare. Whether through stabilizing selection, trait canalization, or due to constraints imposed by complex network interactions, stasis is defined by removal of diversifying trait values. Thus, there appears to be a commonality between, on the one hand, diversification of traits in divergent evolution and, on the other hand, homogeneity of traits in evolutionary stasis. In both cases, certain trait values are suppressed, and the suppressed values are those that substantially deviate from the population mean – either from the mean trait values in the newly formed groups in the divergent dynamics (the intermediate forms) or from the mean trait value of the static population (the “diversifying” forms, Figure 1). The mechanisms driving such suppression may be similar in both dynamic and static evolutionary processes, but they have not been fully elucidated.

### Competitive suppression and exclusion may contribute not only to population dynamics but also to stasis

**Figure 2:**
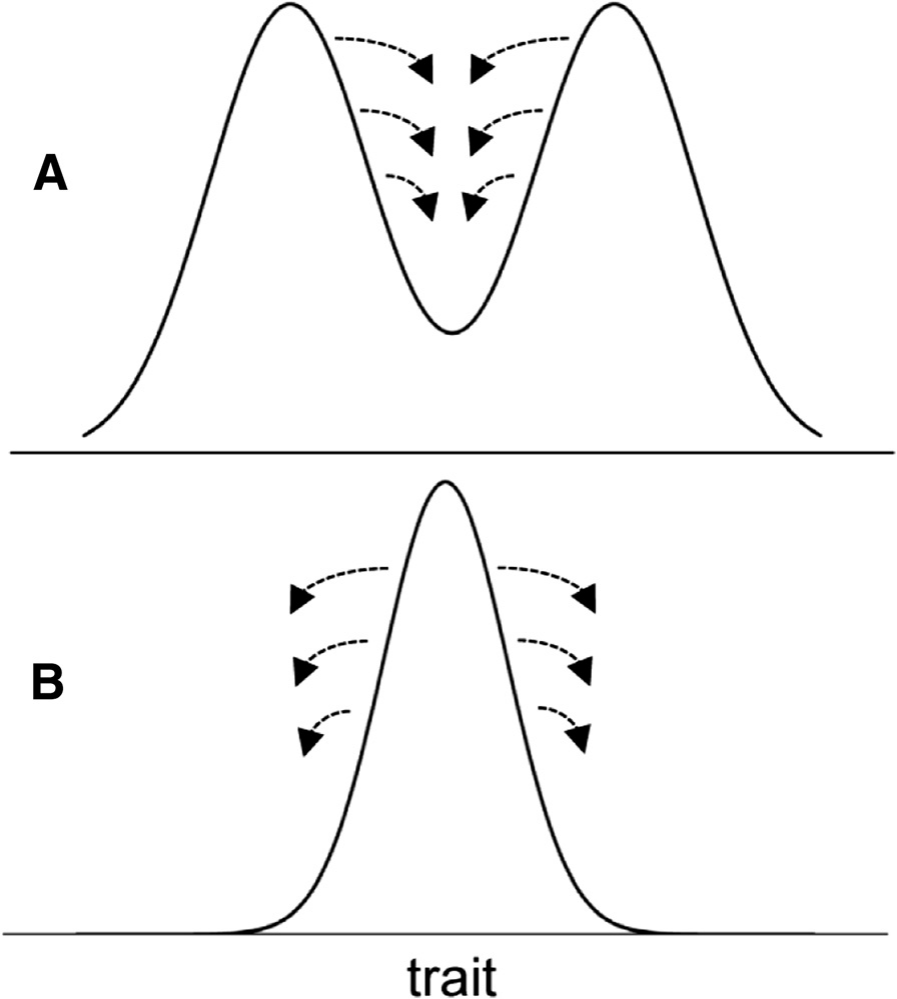
In both diversifying (A) and static (B) populations, competitive pressure from the more successful (i.e., more fit) forms on either intermediate or diversifying forms (arrows) may centrally contribute to population dynamics in combination with environment-driven selection or, perhaps, as the sole mechanistic force.

As stated above, the suppression of outlying trait values – either the intermediate forms in divergent dynamics or the diversifying forms in static populations – is commonly observed, but the trivial explanation that it occurs through direct environmental selective pressure does not appear to fully explain this phenomenon. Darwin himself realized that explaining the lack of intermediate forms by their diminished fitness due to purely environmental influences is not enough and that separate attention should be given to a mechanism stemming from populational interactions, specifically, competitive suppression and, ultimately, exclusion (removal) of certain forms. Darwin focused on elimination of intermediate forms through competition in his argument that “the range of inhabitants of any country by no means exclusively depends on insensibly changing physical conditions, but in a large part on the presence of other species … with which it comes into competition; … each species on the confines of its range, where it exists in lessened numbers, will, during fluctuations in the number of its enemies or of its prey, or in the nature of the seasons, be extremely liable to utter extermination; and thus its geographical range will come to be still more sharply defined.” He then convincingly illustrated his point with an example of artificial selection of three varieties of sheep: the mountain, hill, and plain breeds (see pages 136–137 in [26]). This example of Darwin’s was geographic, but the same logic may be adapted to non-geographic traits. Instead of location (mountain, hill, and plain in Darwin’s example), other traits may be similarly considered, for example, a preference for specific kinds of food, as in the case of Darwin’s finches, or a preference for specific sexual dimorphic features in a partner in the case of sexual selection. The notion common to these geographic and non-geographic cases is that intermediate forms are suppressed through competitive suppression and, ultimately, competitive exclusion (Figure 2A). Similar logic may be applied to static populations, in which trait homogeneity is maintained by intraspecific competition that suppresses diversifying forms (Figure 2B).

There are at least two immediate questions that need to be addressed regarding these considerations. One, how exactly do two units (individuals) with different trait values come into competition? Competition among individuals carrying the same trait values is intuitively understandable, because the same trait value is equivalent to a requirement for the same resources, such as food, space, or mates. However, two individuals with different trait values will presumably be dependent on two different qualities of the resource. Using the well-known Lamarckian example, two giraffes with similar neck lengths will compete for foliage at the same altitude above the ground, whereas two giraffes with different neck lengths will consume foliage at different altitudes and thus should not compete. Therefore, inter- and intra-specific competition leading to suppression of transitional and diversifying forms, respectively (Figure 2), needs a more thorough explanation. Two, can this mechanism of suppression/exclusion be the sole driver of population divergence or static homogeneity without environmental selective pressures, that is, in a homogenous and stable environment? The goal of this study is to answer these questions by considering the mechanistic contribution of the phenomenon of trait degeneracy.

### Trait degeneracy causes distributed, non-local competition for resources and subsequent non-local competitive suppression and exclusion

We propose that the phenomenon known as trait degeneracy centrally contributes to suppression of transitional and diversifying forms in diverging and static populations, respectively. Moreover, we argue that these two modes of suppression are manifestations of the same mechanism. Gerald Edelman, whose pioneering ideas have inspired this entire field of research [28], has defined degeneracy as “the ability of elements that are structurally different to perform the same function or yield the same output” [29]. In other words, degeneracy is in play when the same function is performed by structurally diverse units of an ensemble; that is, each of the individual units is multi-functional. In biology, degeneracy is ubiquitous [28-39]. At the molecular level, numerous enzymes, cell surface receptors, and antibodies have been shown to bind not only their principal substrates, ligands, or antigens but also other molecules that may or may not be similar to these binding partners. As a result, degeneracy is manifested as enzymatic substrate ambiguity, multiple ligand–receptor cross-reactivity, and moonlighting of proteins and protein complexes [34,40]. Degeneracy is also seen in ontogenesis [41,42], the immune [43] and nervous systems [29], metabolic pathways [44], and cell signaling [45]. At the level of heredity, degeneracy is manifested not only in the well-known degenerate nature of the genetic code but also as degenerate genotype–phenotype mapping, when diverse genotypes are manifested as similar phenotypes and identical genotypes are manifested as diverse phenotypes. This functional versatility also enables multiple uses of organs, such as the use of fins for swimming and crawling. At the level of ecosystems, phenotypically diverse individuals in predator species will consume the same prey and, conversely, nearly or completely identical individuals (twins) will consume different kinds of prey. This view can be easily extrapolated to social systems, as individual humans or organizations in a society act degenerately, being capable of performing similar functions despite their obvious diversity [36]. However, functional degeneracy must be clearly separated from the process of degeneration, defined as deterioration of a structure with an accompanying decline or loss of function [39].

**Figure 3:**
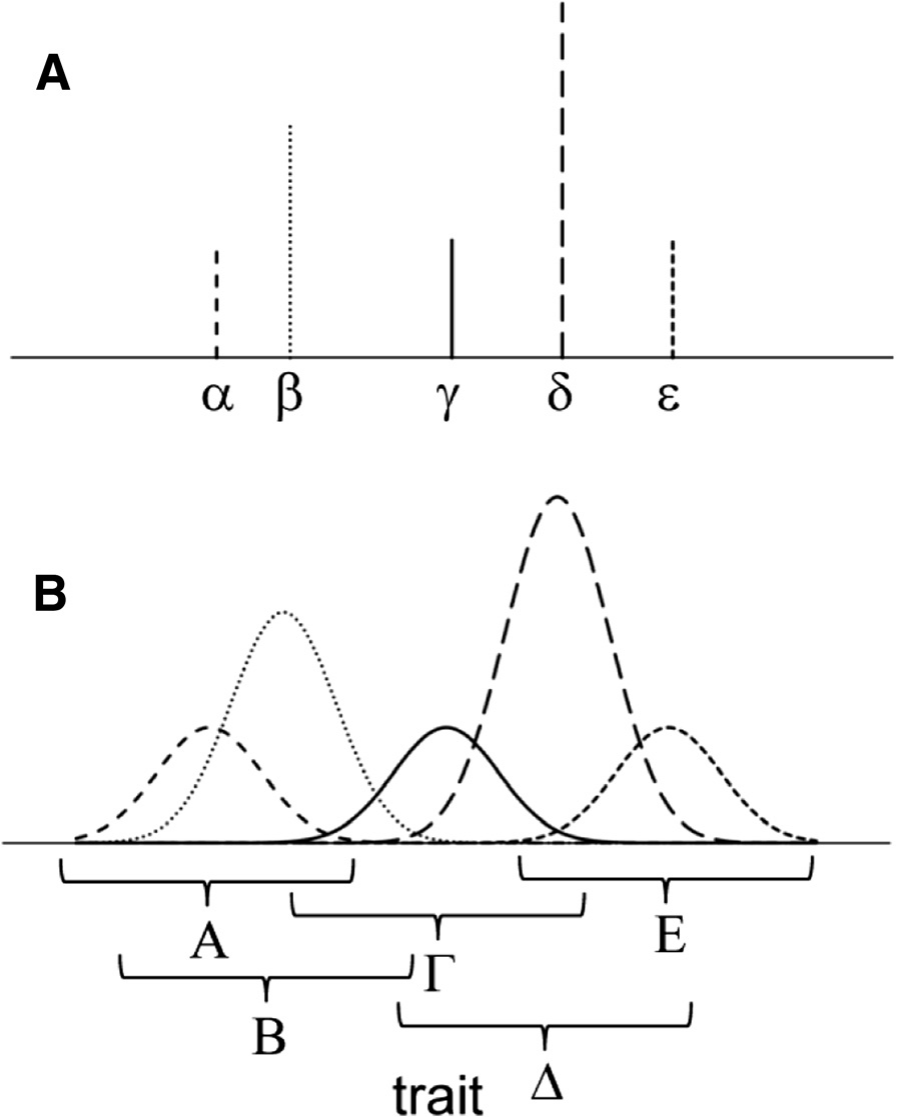
**Schematic representation of diverse (A) and degenerate (B) populations, each consisting of 8 individuals.** In **A**, one individual for each of the **α**, **γ**, and **ε** types, two individuals of the **β** type, and three individuals of the **δ** type are shown, with each type characterized by specific trait values. In **B**, by contrast, types are represented not by trait values but by ranges of values, manifesting degeneracy. In this hypothetical case, the degeneracy function is Gaussian. The area under each of the curves **Α**, **Γ**, and **Ε**, representing three different types, is 1. The areas under **Β** and **Δ** are 2 and 3, respectively, representing 2 and 3 individuals of each type in this small hypothetical population. Note the overlap of the degeneracy ranges. In this case, the degeneracy ranges are equal for all types and individuals, but this is not required in a more general case.

Degeneracy is an intermediate state between diversity and redundancy. In degeneracy, diversity is present, but each of the diverse elements is not perfectly unique and is partially similar to other elements of the group. Redundancy is also present, but, unlike completely redundant groups, the elements are not exact copies of each other and partially differ. Degeneracy may also be defined as functional micro-diversity of individual elements in a group or population so that structurally unique elements of repertoires function similarly to other elements. In biological populations, genotypes are diverse, but phenotypes, through their functional flexibility, are degenerate [34]. A simpler definition of degeneracy is “limited functional sloppiness” of the elements of structurally diverse populations [33]. Figure 3 illustrates the relationship between diversity and degeneracy. As shown for the case of diversity, individuals are characterized by their locations on the scale of trait values, whereas in the case of degeneracy, ranges – not points – on the scale characterize the individual units in a population. Therefore, degeneracy is represented by a *population* of functional responses manifested by an *individual*. Of critical importance, the individual ranges of degeneracy can overlap, suggesting that the degenerate – not exactly similar and not completely different functionally – units will compete for the resource in the overlapping regions. Realistic degenerate populations are much larger than the simplified case shown in Figure 3, with hundreds to billions of overlapping individual degeneracy ranges forming a continuous spectrum of trait manifestations [29-36,40,41].

Degeneracy is a fundamental feature of biological systems, which is centrally important for their reliability and adaptability and, according to Edelman, “both necessary for, and an inevitable outcome of, natural selection” [28-35,39]. Degenerate repertoires are reliable because the loss of an individual element will have only a small impact on the whole repertoire, due to the overlapping functionality of multiple elements. Thus, the function of a missing element will be performed by other units, which, although somewhat different, may still function similarly to the missing unit. Degeneracy critically adds to the value of diversity in ensuring population survival in novel, previously unexperienced environments. Biological systems are notorious for successfully dealing with novelty to which they could not have possibly been adapted through previous selection. Life on Earth has adapted to the most extreme conditions and to every change in global and local environments. The immune system elicits effective protective responses even to new antigens that have been artificially synthesized in the laboratory and have not occurred naturally before. The nervous system adapts to (learns) the most complex information flows. In all cases, it appears that the selective repertoires “know what to expect” in advance and successfully react to whatever unpredicted novelty is encountered. Diversity alone cannot be the answer to this paradox. No matter how diverse a repertoire, the unpredictability of the future is larger, and therefore any new selective challenge from the environment has the potential of generating no adaptive response by any member of the repertoire. In this case, there will be no subsequent selection and no selection-based adaptation – the population as a whole will become maladapted and, likely, extinct. However, in a degenerate population (e.g., the adaptive immune system, the brain, and evolution), the “fuzzy” nature of the functionality of the population traits drastically raises chances that there will be a unit, or, perhaps, several units, which will respond, even if imperfectly, to the unpredictable new changes in the environment. This unit, or units, will thus survive and form the basis for future selection-based adaptation to the new environment. Considering again the Lamarckian giraffes, in the case of strict diversity, every animal would be able to consume foliage at its individual height, but not above or below it. If a herd of giraffes wanders into an area where foliage is abundant at the heights not matching individual animals, the majority of the herd will starve despite the apparent diversity of the repertoire. In reality, however, this does not happen, because an animal can reach somewhat below or above its height. The repertoire of heights in the herd is degenerate, not just diverse. Thus, the foliage at any height within and even somewhat outside the spectrum of heights is accessible, ensuring survival. A population that is diverse is thus less perfectly adapted than a degenerate population. This simple example illustrates a universal principle that applies not only to species but also to cells, molecules, and social structures [28-39].

The central role of degeneracy is directly supported by Mason’s reasoning on the “Cambrian explosion” [39]. Referring to Budd’s works [46,47] and Budd’s understanding of “redundancy arising from shared function (functional degeneracy)” [46], Mason argues that the rapid development of phyla during the Cambrian was strongly linked to degeneracy, and that we need to be “enquiring how the cellular machinery during this period was highly degenerative” [39].

Previously, we have pointed out that, in addition to these obvious benefits of trait degeneracy for populations, it adds one more feature, distributed competition for resources [30,32-35]. This degeneracy-driven competition contributes to the self-patterning of degenerate asexual populations [30,32-35] (although, as argued in Discussion, the results are likely applicable to some sexually reproducing populations). Here, we develop this notion further, demonstrating, in an analytical model and in a cellular automata-based computer model, that competition due to trait degeneracy drives both dynamic diversification and static homogeneity in populations that exist in a stable environment, which is modeled by a stable resource distribution. In other words, the results described below show that the intrinsic population dynamics stemming from suppression of certain forms by competitive pressure arising from trait degeneracy may drive either divergence or homogeneity of populations, without a requirement for environmental change.

## Theoretical considerations

To estimate the possible effects of degeneracy on populations, consider a simple one-dimensional population in which individuals are characterized by a single trait. The population is diverse, with individuals differing in, and definitively characterized by, their specific trait values. The trait defines the ability to consume a diverse resource, which can also be plotted on the same scale of values. For example, a plant foliage resource is distributed above the ground—short, medium, and tall grasses, short, medium, and tall bushes, and short, medium, and tall trees. Correspondingly, herbivorous animal populations are distributed on the same scale, from small rodents to giraffes. Such distributions do not have to be merely spatial. For example, one can plot a distribution of small prey (small mammals, insects, etc.) according to how fast they escape from predators (e.g., larger carnivorous animals). The predators will then be distributed according to their average speed in chasing prey. In a similar fashion, one could distribute all antigens and their corresponding antigen-specific recognizers (antibodies or T cell receptors).

**Figure 4:**
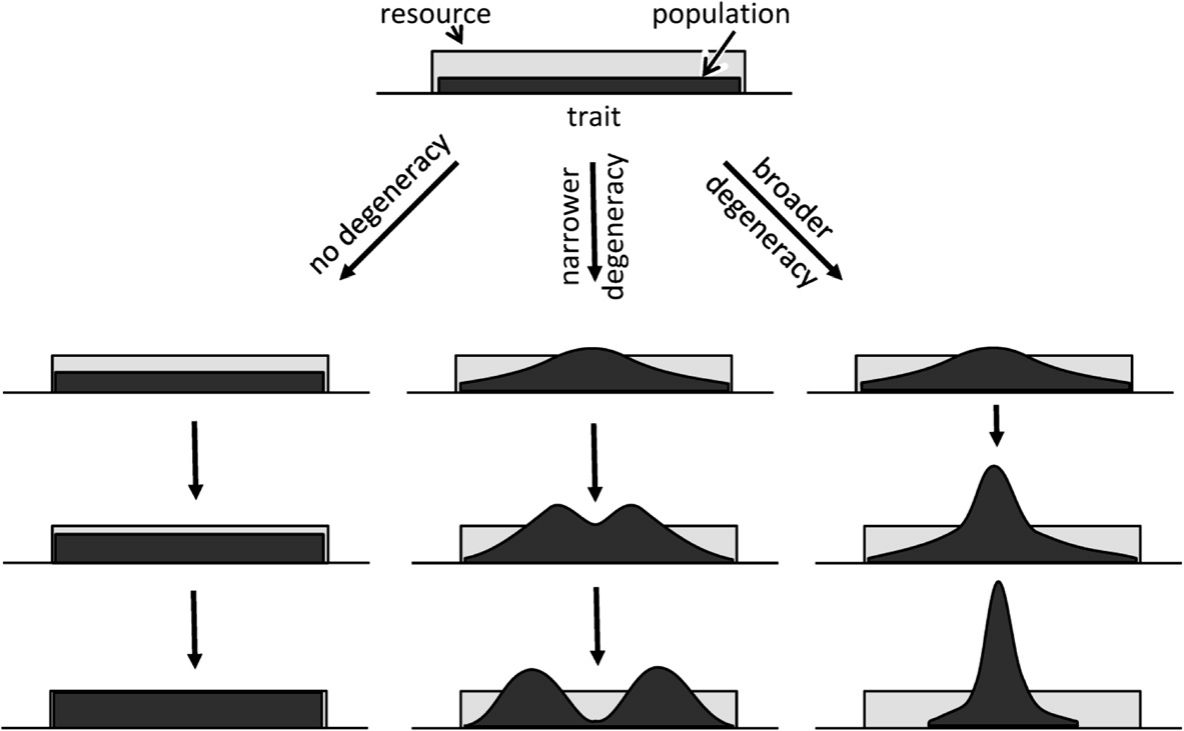
Predicted dynamics in a hypothetical population placed in a continuous and uniform environment represented by a rectangular distribution of resource under the scenarios of no trait degeneracy (left), narrow degeneracy (middle), and broad degeneracy (right).

Realistically, any distribution of resource has limits. In the foliage distribution example, there are obvious limits to how short or tall plants can be. Consider a simple rectangular resource distribution within a certain range and a population that is also evenly distributed on the same axis according to the trait values (Figure 4). In the absence of degeneracy, individuals in each location will compete among themselves for the resource, but not with individuals at other locations. Therefore, the subpopulations at each of the trait values will grow independently of the growth of other subpopulations (Figure 4, left branch). However, in realistic populations, individuals manifest trait values that are not simply diverse – they are degenerate, as discussed above, and are defined not only by locations but by ranges on the trait axis. In this case, the location of an individual on the trait axis is defined by its median trait value.

### Narrow degeneracy

Starting with the same initial population, different dynamics would be expected depending on the type of degeneracy. Consider a population in which the range of trait degeneracy is substantially narrower than the distribution of resource (Figure 4, middle branch). The individuals in the middle of the distribution have access to much wider resource ranges than the individuals on the outskirts of the distribution. Therefore, the growth rates will be higher for the subpopulations in the middle of the distribution, and these subpopulations will approach the carrying capacity of the resource faster. Simultaneously the competitive pressure in the middle of the resource distribution range will increase not only among the individuals with the same median trait values but also among individuals with similar, adjacent values due to trait degeneracy, which is an example of non-local, distributed competition. At some point, the competitive pressure in the middle of the distribution becomes the highest, because this location designates the kind of resource that is accessible, due to trait degeneracy, to the majority of individuals in the population. The limited resource in the middle of the distribution is no longer able to sustain growth rates that are necessary to compensate for mortality from the increasing competitive pressure. As a result, the subpopulations in the middle of the distribution manifest “negative growth,” with the increasing gap in the formation of a bimodal (or, perhaps, multimodal, depending on how narrow the degeneracy spread is) distribution of the population. The separating parts of the distribution can be viewed as newly emerging species, which move apart to minimize the non-local competition defined by their trait degeneracy. There is a limit, however, to how wide the split in the distribution will become, because there is no resource outside of the distribution range, even though the individuals that are more peripheral to the overall density distribution could, due to trait degeneracy, reach outside of the resource distribution range. In the end, the competitive pressure in the middle splits the distribution into two (or more) parts (Figure 4, middle branch). The “transitional” subpopulations located between the newly formed species experience the highest competitive pressure from both sides, and will ultimately be completely excluded. Importantly, the divergent dynamics in this example take place in the continuous environment represented by the rectangular resource distribution. This may be viewed as a case of sympatric speciation, which occurs without geographical isolation of the newly forming species. Moreover, the population divergence in this case is not directly driven by the environment, which remains static and uniform across the distribution range. Instead, the predicted dynamics are driven by non-local competition, which is distributed, due to degeneracy, across the entire range of trait values. These considerations apply to any degenerate selective repertoire. Universally, degenerate populations with degeneracy ranges that are narrower than the resource distribution are expected to be divergent. These theoretical considerations have been supported by the results of computer modeling utilizing the cellular automata approach [30,31] and in an analytical model [35] and are discussed in more detail elsewhere [32-34].

### Broad degeneracy

Next, we analyze, for the first time, the case in which the degeneracy range is broad enough to be comparable with the resource distribution range (Figure 4, right branch) and its relation to the diversifying dynamics that have just been considered. As in the case of narrower degeneracy, the individuals in the middle of the distribution will propagate faster, because they attempt resource consumption outside of the resource distribution less frequently than the individuals on the periphery. The higher growth rates in the middle of the distribution will not, however, exhaust the carrying capacity of the resource as quickly, because broad degeneracy allows for resource consumption by the centrally located individuals across almost the entire range. Thus, in the middle of the distribution, less intense competitive suppression with no extinction is expected. Unlike the case of narrow degeneracy, broad degeneracy allows the rapidly growing central subpopulations to exert stronger competitive pressure on and rapidly suppress the peripheral subpopulations. The suppression of the latter is furthered by their broad trait degeneracy, because the individuals with more extreme trait values attempt resource consumption outside of the distribution range more frequently. Since no resource is available outside of the distribution range, such attempts will be fruitless, thus putting these peripheral elements at further disadvantage. As a result, continued growth in the middle of the distribution with exclusion of peripheral subpopulations is expected, and the overall population will increase its trait homogeneity around the median values.

Importantly, such an increase in homogeneity is propelled not by the environment as such, but by the interplay between broad degeneracy and the environment. The environment represented by the resource distribution is uniform and homogenous across the entire range. It does put some centrally located individuals at a certain advantage, whereas the more peripheral individuals are less efficiently positioned for finding the resource due to frequent attempts to seek resource outside its distribution range (due to degeneracy). However, in the absence of non-local, degeneracy-driven competition, local competition would occur only among the individuals with identical trait values (local competition), and the growth of peripheral subpopulations would ultimately catch up with that of the central subpopulations. These considerations suggest that the frequently observed static trait homogeneity [1-8] may be driven mostly by non-local (defined by broad trait degeneracy) competition for resource. We propose that this mechanism centrally contributes to evolutionary stasis, which cannot be explained solely by stabilizing pressure from the environment [9].

## The Model

### Analytical version of the model

The model is a one-dimensional “universe” represented by space *S* of a single characteristic, or trait, of cellular automata (also referred to as “units,” “individuals,” or “organisms”) containing a resource that these organisms require for survival and propagation. Reflecting the finite amount of resource in this Verhulstian model, the carrying capacity *K* is distributed in *S* according to *K*(*s*), where individual locations *s* ∈ *S*. Organisms are also distributed in the same space according to *N*(*s*). In this generalized model, *S* may be (but does not need to be) a geometric space. *S* may represent non-geometric traits, such as antigenic properties of immunogens, a particular quality of food, or the receptor-binding characteristic of a ligand. *S* may also represent physical space in which case *K*(*s*) could be a distribution of vegetation (food for herbivores) along a river bank or at various altitudes on a mountain. In a specific case, *K* may represent the amount of livable physical space for the organisms at a given location *s*. The only characteristic of organisms and of resource units in this model is their individual location.

In a trivial version of this model with strict specificity of resource consumption, the relationship between units and resource is defined by their individual locations. If the location of a unit is the same as the location of resource, the unit will attempt consumption of that resource. If their locations differ, consumption of resource is impossible. Thus, population dynamics *N*(*s*_0_, *t*), where *t* is time, at any location *s*_0_ will be independent of any other location in *S*, and the dynamics of the overall population of organisms will be characterized by a series of mutually independent logistic growth curves, each at a separate *s*:

(1) $$\frac{\mathrm{dN}\left(s_{0}\right)}{\mathrm{dt}}=\mathrm{rN}\left(s_{0}\right)\left(1-N\left(s_{0}\right)/K\left(s_{0}\right)\right),$$

where *r* is the Malthusian growth rate. Since the units are characterized solely by their location, *r* is location-independent and universal for all locations and thus is a characteristic of the system rather than of subpopulations at various *s*. The more general case of location-dependent *r*(*s*) is not considered here.

The purpose of our model, however, is to analyze the effect of degenerate resource consumption on population dynamics. In this more complex version, a unit located at *s*^′^ consumes resource degenerately across *S*, so that the maximal resource consumption will still occur at *s*^′^, yet consumption is also possible at other locations *s* according to the degeneracy function *f* (*s*, *s*^′^), a unimodal kernel function, which is non-negative, piecewise continuous, even (so the absolute value notation is dropped), integrable, and normalized; that is, ∫_*S*_ *f* (*s*)*ds* = 1. In the specific implementations of the model discussed below, the Gaussian function was chosen to approximate degeneracy, that is, $f\left(s\right)=\exp\left(-s^{2}/2\sigma^{2}\right)/\sigma \sqrt{2\pi }$, where *σ* defines the “degeneracy range” and is referred to as such below.

For a single subpopulation at *s*^′^ in the absence of units at any other location, the carrying capacity will increase due to such degenerate resource consumption and will become *K*_*s*'_ = ∫ *f* (*s*, *s*′)*K*(*s*)*ds*. For simplicity of notation, the integration here and below is taken over *S* unless otherwise indicated. As stated above, the individuals in the population are characterized by a single trait, which is their preferred location for resource consumption and defines their own location in *S*. With this consideration, the units do not differ in their ability to consume resource degenerately, meaning that the degeneracy function does not depend on the absolute values of *s* and *s*^′ ^and will be a characteristic of the system as a whole. This consideration leads to an important simplification: the degeneracy function is universal for all units in the system, is location-independent, and depends only on the distance between the organism and resource, *f* (*s*–*s*^′^) (assuming a “symmetrical” degeneracy function). The more general case of location-dependent *f* (*s*, *s*^′^) is not considered here. In an even more general case, also not considered here, different units at a given location may differ in their degeneracy. The dynamics of such an isolated subpopulation with degenerate resource consumption will then be:

(2) $$\frac{\mathrm{dN}\left(s'\right)}{\mathrm{dt}}=\mathrm{rN}\left(s'\right)\left(1-N\left(s'\right)/\int f\left(s-s'\right)K\left(s\right)\mathrm{ds}\right)$$

Realistic populations, however, are distributed in *S*, meaning that more than one subpopulation is present and that any two units located at *s*^′^ and *s*^′′^ may compete for resource at *s* due to degenerate resource consumption. Thus, degeneracy leads to a new kind of competition. In addition to competition among similar units, various subpopulations now compete for resource at various locations through degeneracy. The latter phenomenon needs formal description.

Consider consumption of resource located at *s*_0_ by all units of the population. In order to perform this analysis, we will utilize the notion of “demand,” defined as the amount of limitless resource that would be consumed in the absence of any competition. The demand for resource at *s*_0_ by units at *s*^′^ will be proportional to *f* (*s*_0_ − *s*^′^)*N*(*s*^′^), whereas the overall demand for resource at *s*_0_ by the entire population will be proportional to ∫ *f*(*s*_0_ − *s*^′′^)*N*(*s*^′′^)*ds*^′′^. Due to the stated equality of the competitive abilities of the units, the total carrying capacity at a location *s*_0_, *K*(*s*_0_), will be distributed among various consumers according to their demand for that resource. The fraction of de mand for resource at *s*_0_ by a subpopulation *N* (*s*^′^) in the overall demand for that resource is $f\left(s_{0}-s'\right)N\left(s'\right)/\int f\left(s_{0}-s''\right)N\left(s''\right)ds''$. Then, the carrying capacity at *s*_0_ that is available to *N*(*s*^′^) is Ks',s0=Ks0fs0−s'Ns'∫fs0−s''Ns''ds''. This allows for re-writing equation (2) for the degenerate resource consumption by the entire population:

(3) dNs'dt=rNs'1−Ns'/∫Ksfs−s'Ns'∫fs−s''Ns''ds''ds

Finding solutions of this differential-integral equation analytically would require non-trivial approaches. Therefore, we utilized numerical (Runge–Kutta 4^th^ order) integration to find the solution.

### Cellular automata-based approach

Classical models can be adopted to account for degenerate resource consumption, and they show interesting dynamics, but solving such degenerate models analytically is challenging [35]. Also, analytical solutions need to be rederived with each modification of a model, resulting in additional burden. Therefore, to complement the analytical model (3), we used a simpler approach to model degenerate repertoires as populations of cellular automata [30,31].

In the cellular automata-based version of this model, the distribution of resource is represented by a one-dimensional array of discrete values. The size of the array represents the spread of the one-dimensional trait space, the index of each element represents a specific trait value, and the value of each element represents the carrying capacity at that location. The population is represented by a set of cellular automata, each characterized by its location (index) similar to the resource above, whereas the trait degeneracy range is universal for all automata. The flow of time is cyclical, represented by iteration cycles. Each automaton (individual) is processed at each cycle, with the important caveat that the order of processing is randomized at every cycle. The resource is “consumed” by cellular automata during a cycle, but is restored to the initial state at the beginning of each cycle. During each cycle, each cellular automaton:

• expends a constant fraction of previously consumed resource for self-maintenance (“cost of living”);

• attempts to feed (consume resource) at a location that is randomly selected using a Box-Muller Gaussian random number generator, with *σ* defining the degeneracy range around the automaton’s location; if resource is available at the degenerately selected location, a constant unit of resource is consumed (carried over from the resource array to the automaton);

• if the balance of accumulated resource (the result of expenditure and consumption above) becomes equal or less than zero, the automaton “dies” and is no longer considered;

• if the balance of accumulated resource exceeds an established constant threshold, the automaton “self-propagates,” creating a copy of itself.

This implementation is similar to the previously reported approach [30,31].

## Results

To preserve the focused scope of this study, several specific limitations were imposed: only a Gaussian degeneracy function was considered, with *σ* representing the degeneracy range; the value of *σ* was the same for all locations *s* and did not change with time; and a rectangular static resource (carrying capacity) distribution was analyzed. All implementations of the model have been validated by testing the case of degeneracy *σ*=0, with the results showing the trivial solution – a series of identical independent logistic growth curves as predicted in Figure 4 in the left track. Both numeric differentiation and cellular automata approaches showed similar results for all conditions, with selected cases depicted in the following figures.

### Validation of theoretical considerations for ideal populations

**Figure 5:**
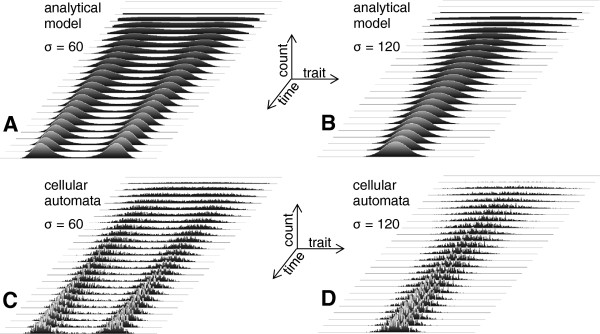
**Overall dependence of population dynamics on trait degeneracy in a stable, homogenous environment defined by rectangularly distributed resource in the range [0, 299].** Solutions of the analytical (**A**, **B**) and cellular automata (**C**, **D**) model show that narrower trait degeneracy (*σ*=60) leads to divergent dynamics with competitive pressure from the formed subpopulations eliminating the transitional forms between them, whereas broader trait degeneracy (*σ*=120) causes phenotypic stasis with continued elimination of the outlying forms and subsequent increasing homogeneity of the population.

Initial simulations considered the cases of narrow and broad degeneracy values (relative to the dispersion of the resource distribution as discussed above; see Figure 4 and related text) in the models described above. Figure 5 shows the results obtained with a rectangular distribution of resource in the range [0, 299] and an initial rectangular distribution of population in the same range. Similar to previous results [35], dichotomous dynamics was observed for relatively low degeneracy values (the result for *σ*=60 is shown in Figure 5A). As theorized above and elsewhere [35], for narrower degeneracy, the forming of “new species” suppresses the transitional forms with intermediate trait values. Using this model, previously unexplored dynamics were observed with broader degeneracy values, such as *σ*=120, as shown in Figure 5B. Instead of dichotomous dynamics, the population narrowed down to the trait values in the middle of the resource distribution, and the population homogeneity continuously increased with time. These dynamics are in agreement with theoretical considerations regarding broader degeneracy, as discussed above, and they have not been explored before as a means to explain evolutionary stasis. Modeling with cellular automata again confirmed the abovementioned theoretical considerations and previous modeling results [30,31] for narrow degeneracy (the result for *σ*=60 is shown in Figure 5C). As expected for narrower degeneracy, the cellular automata model also shows dichotomous population dynamics, with the forming of “new species” suppressing the transitional forms with intermediate trait values. Using this model, we then analyzed, for the first time, broader degeneracy values, such as *σ*=120, as shown in Figure 5D. Again, in agreement with the theoretical considerations above, the population narrowed down to the trait values in the middle of the resource distribution, and the population homogeneity continuously increased with time. Therefore, we conclude that broader trait degeneracy promotes evolutionary stasis, whereas narrow degeneracy promotes divergence. Importantly, certain trait values (transitional in the dichotomous cases and outlying in the cases of increasing homogeneity) are suppressed by degeneracy-driven distributed competition for resource. It appears that both evolutionary divergence and stasis are propelled by the same degeneracy-driven mechanism, and the superficial difference between them is defined solely by the relative dispersion of resource distribution and degeneracy.

**Figure 6:**
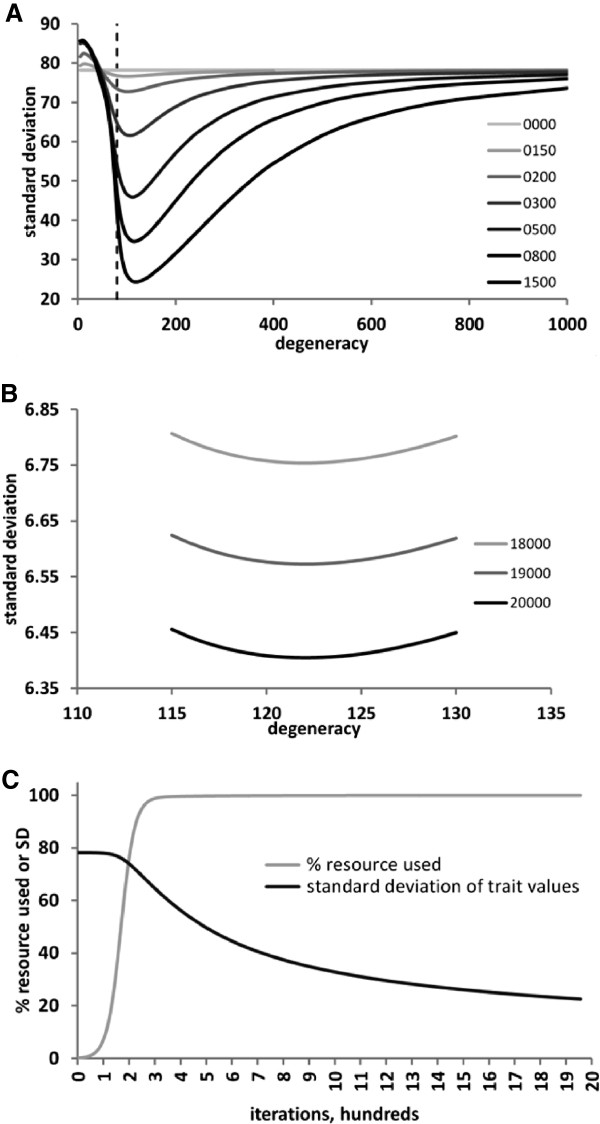
**Dependence of population dynamics on broader trait degeneracy in a stable, homogenous environment at lower (A, C) and higher (B) iterations of the solution of the analytical model.** In **A**, the relationship between degeneracy value *σ* (horizontal axis) and standard deviation of the trait values in the population (vertical axis) is shown by curves of various shades of grey color for the indicated iterations of the numerical solution of equation (3). The vertical dashed line shows the separation between dichotomous (on the left) and stabilizing (on the right) dynamics. Trait homogeneity of the population continues increasing with time, as shown for greater iterations in panel (**B**). Importantly, the narrowing of population diversity continues after the overall population size has reached its maximum and the entire available resource has been consumed (**C**). Therefore, the increasing homogeneity of the population is not driven by adaptive maximization of resource exploitation.

Using this model, more detailed analyses were then performed for the first time for prolonged homogenous population dynamics with broader degeneracy (Figure 6). The homogeneity of the population was measured by calculating the standard deviation (SD) of all individual trait values, considering that the population appears near-normally distributed (Figure 5B, D). The smaller the SD, the narrower the distribution of the trait values, and the more homogeneous the population. Figure 6A, B demonstrates that population homogeneity was dependent on degeneracy: in the case of a rectangular distribution of resource in the range [0, 299], the SD of trait values was the smallest at *σ*=122. Lesser degeneracy values caused dispersion of the population, leading to the dichotomous dynamics for *σ* values on the left side of the vertical dashed line in Figure 6A. Greater degeneracy values also broadened the distribution of trait values but without divergent separation of subpopulations. Wide degeneracy prevents divergent separation, because every individual competes with essentially every other individual in the population, thus eliminating the effect of trait variability and making the population nearly homogenous in its competitive success across the trait values. Importantly, the increasing homogeneity of the trait values did not affect the overall efficiency of the population in consuming resource. The overall fraction of resource consumed by the population increased logistically and rapidly saturated the entire resource being consumed, whereas population homogeneity continued increasing (SD decreasing, Figure 6C) to a phenotypically narrow, highly homogenous population (Figure 6B). This observation suggests that increasing phenotypic homogeneity of evolutionarily static populations may be unrelated to species adaptation and propelled not by selective pressure toward more efficient resource exploitation but by the intrinsic degeneracy-driven distributed competition for resource. This finding is the main result of this study.

That results were similar between the analytical and cellular automata-based versions of the model is important. The latter model is, by definition, stochastic: every element of the population selects resource location degenerately as a normally distributed random value around its own location, yet the overall dynamics of the population remains the same as in the analytical model. Furthermore, the solution was independent of the initial population distribution, including the random initial distribution. Moreover, adding a random chance of dying for each element at each cycle did not affect the solution. Thus, the solution is stable, despite a certain degree of environmental stochasticity.

### Non-local effect of the environment

**Figure 7:**
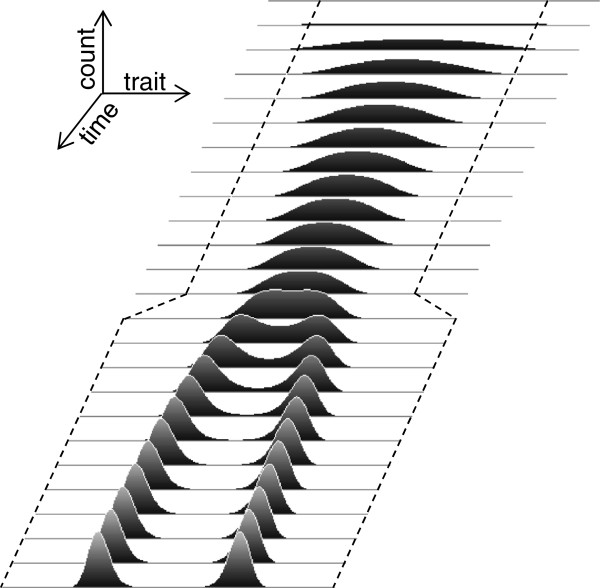
**Non-local effect of a change in resource distribution on population behavior.** The edges of a rectangularly distributed resource are indicated with dashed lines along the time axis. The broadening of resource distribution causes a transition from increasing homogeneity in a phenotypically static population to divergent dynamics. Resource distribution range [0, 299], degeneracy *σ*=55, integration interval 0.025, and a series of evenly time-spaced population snapshots are shown for a total of 3,000 iterations.

The observed modeling results (Figures 5 and 6) confirmed theoretical considerations (Figure 4) that the apparently different behaviors – divergent dynamics and homogenous stasis – are not defined by the environment (which is homogenous and static in this model) but by a common mechanism, degeneracy-driven, non-local competition. A specific outcome depends solely on the relationship between resource distribution and degeneracy, and this relationship affects the entire population; that is, the effects of environment and degeneracy are non-local. To further confirm that this is indeed the case, the model was modified to include a change in the environment (Figure 7). In the same analytical model, the initial resource distribution was narrower for the first half of the solution, and then widened on both sides, leading to an apparent switch from phenotypic stasis with increasing homogeneity to divergent dynamics (Figure 7). Importantly, the broadening of resource diversity was relatively small (e.g., by 1/6 of the overall range on both sides of the distribution in the example shown in Figure 7), but its impact on the system’s behavior was pivotal. Moreover, although the change occurred on the edges of the resource distribution, the population changes occurred in the middle. This result confirms that the distributed, non-local nature of the competitive interactions stemming from phenotypic degeneracy drives both evolutionary stasis and divergent dynamics. This result also suggests that the observed sudden transitions between evolutionary stasis and speciation [8-17] may be driven by subtle changes in the environment that are remote from the mainstream phenotypic characteristics of the population.

### Incorporating variability of heritable phenotypes

**Figure 8:**
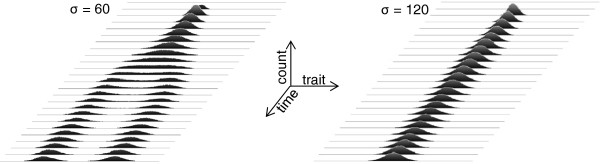
**Stability of the model solutions despite heritable phenotypic trait variability.** Cellular automata-based implementation of the model is shown. In each case, a single founder individual was placed in the middle of the tested range [0, 299] of trait values. Both divergent dynamics (left) and stasis (right) are preserved for the indicated degeneracy values (*σ*) despite trait mutability, which was 2 in both cases. Similar overall results were observed with higher tested mutability values (up to 15), but a further increase in mutability tended to overwhelm the effect of degeneracy, leading to a more homogenous population broadly distributed across trait values.

Phenotypes vary, providing the substrate for natural selection. In the model under consideration, adding such variability is equivalent to the offspring differing from their parents in the axis location of their trait values. To assess the possibility that such variability may affect the behavior of the system, a modified cellular automata-based model was tested in which offspring locations were normally distributed around a parent’s location, with the dispersion (standard deviation) of such a distribution denoted as “mutability.” Due to mutability, an initial population represented by one or a few individuals spreads with time across the spectrum of trait values (Figure 8). This version of the model represents the development of new populations from much smaller and less diverse founder colonies. Considering that natural heritable variations are usually substantially smaller than phenotypic flexibility, mutability values smaller than the degeneracy were tested to preserve the biological relevance of the model. Despite heritable mutability within a certain range of values, the degeneracy-driven competitive interactions preserved behaviors similar to those in the absence of heritable mutability: narrower degeneracy values promoted divergent dynamics, whereas broader values promoted phenotypic stasis (Figure 8). At higher mutability values, the effects of degeneracy were diminished, and the population tended to distribute more homogenously across the entire spectrum of trait values. These results prove that the solution is stable not only despite a certain degree of environmental stochasticity (see above), but also despite limited heritable stochastic variability.

## Discussion

We investigated a model of degenerate resource consumption in diverse populations placed in a stable homogenous environment. Analytical and cellular automata implementations of the model have been tested. The results suggest that degeneracy of a heritable phenotypic character – a preference for a particular kind of resource – leads to distributed competition for resource. This degeneracy-driven distributed competition facilitates either divergent dynamics or homogenous stasis, depending on the relative dispersions of trait degeneracy and resource distribution. The solutions are stable in the presence of limited environmental stochastic variability or heritable phenotypic variability. Importantly, the observed behaviors – either heritable phenotypic divergence or stasis – did not change the overall adaptation of the population in that they did not affect the total population size or the overall efficiency of resource consumption.

Such a role of degeneracy in population dynamics was not initially predicted in the early works that were concerned with the contributions of degeneracy to reliability in stable environments and adaptability to novel environments [28,29]. In teleological terms, the “purpose” of degeneracy is to increase fitness. In a stable environment, degeneracy ensures the ability of a population – of individuals, cells, or molecules – to withstand a significant structural loss without a long-lasting functional defect. Such robustness is possible, because, in a system composed of degenerate units, the function of the damaged or lost elements is taken over, due to functional degeneracy, by the remaining elements. Such degeneracy-dependent robustness is critical for survival in familiar environments [28,29]. Arguably, without degeneracy, trait diversity alone would not be able to safeguard the survival of populations following drastic changes in the environment: all current populations have been shaped by past natural selection, which leaves populations unprepared for novel environmental challenges. No matter how diverse a population, the diversity of novel environmental features is larger, and survival due to an increase in diversity alone cannot be ensured. In addition to trait diversity, degeneracy is needed, because it drastically increases the spectrum of possible adaptive responses to novelty [28,29]. The degeneracy-driven dynamics observed in our model have no direct adaptive benefits to a population as a whole. Indeed, in a stable, homogenous environment represented by uniform resource distribution, the shape of the population distribution varies without affecting overall fitness. As such, the observed “patterning” of populations is an “unintended” (using the same teleological terminology) consequence of degeneracy. Nevertheless, the observed dynamics would contribute to both divergent evolution and static homogeneity in populations.

There are several implications to these findings. One, these results add specifics and significantly expand Darwin’s explanations to the difficult question, “why do species exist?” By considering the contribution of degeneracy-driven competition, we add important details to Darwin’s argument for the role of intra- and inter-population rivalry in suppression and, ultimately, exclusion of intermediate forms. Two, our results suggest that the same mechanism plays a key role not only in the emergence of diversification, but also in maintaining population stasis. In both cases, an environmental change is not required, and the population behavior – either divergence or stasis – is defined solely by degeneracy in relation to the overall environment. Three, these findings suggest that observed rapid (in evolutionary terms) transitions between prolonged evolutionary stasis and rapid diversification [1-8,10,11] are due to the overall (non-local) broadening or narrowing of resource distribution, leading to diversification or stasis, respectively.

An important limitation is that only asexual reproduction has been modeled, as the “offspring” are exact copies (except the version with phenotypic variability as shown in Figure 8) of the “parent” (based on their identical location in the model space). This is, in part, a result of the reductionist nature of this model and, in part, a consequence of heritable phenotypes rather than genotypes being considered. Phenotypes but not genotypes are the primary substrate of selection, and we needed to avoid the considerable complexities associated with variable genotype–phenotype mappings [48-50], which is a topic beyond the scope of the current work. Furthermore, investigating the effects of sexual reproduction in degenerate repertoires will require a separate investigation, owing to the need to assess the contributions of complete and incomplete dominance, co-dominance, and epistasis, not only on median trait values but also on degeneracy itself, as the latter may vary among individuals. Clearly, our model does not consider hybrid speciation, and it can be readily envisioned that heterospecific mating would abrogate the observed patterning of model populations. Exchange of genes between organisms belonging to divergent lineages and thus leading to “reticulate evolution” has been well documented [51,52]. Even less remote (conspecific) crosses between phenotypically distant individuals (negative assortative mating) might significantly attenuate the model dynamics. Therefore, the effects of sexual reproduction will be studied in our future work.

Nevertheless, it can be argued that the results of this modeling, particularly the version with phenotypic variability (Figure 8), may be relevant not only to asexual but also, in part, to sexual reproduction. Our findings remain relevant to reproductive isolation leading to conspecific crosses between phenotypically similar individuals in sexual species. Reproductive isolation occurs through behavioral (trait-based positive assortative mating), morphological, physiological, or biochemical mechanisms. The research literature abounds with evidence of reproductive isolation, including positive assortative mating (reviewed [53,54]). It can be argued that in these cases, the crosses are rather similar to the parents, as mate selection occurs from a pool of similar individuals. Even with some randomness in mate selection from the restricted pool, and the subsequent limited stochasticity in the heritable phenotypes of offspring, the overall model dynamics remain preserved, as demonstrated in Figure 8 and described in Results.

Furthermore, recent findings [55] suggest that limited heterospecific hybridization may be also relevant to the results of our model, particularly those in Figure 8. Neotropical primates *Alouatta palliata* and *A. pigra*, which diverged from a shared progenitor about 3 mya, are mostly allopatric, except for a small sympatric area of contact in which they interbreed. In this contact area, purebred individuals coexist with first-generation, backcrossed, and multigenerational hybrids. Assessing morphological variation of genetically confirmed hybrids, the researchers found that “multigenerational backcrossed hybrids resemble the parental species with which they share most of their alleles.” In contrast, “intermediate hybrids exhibited great variation in morphology,” but such intermediates comprised only ~12% of the individuals in the hybrid zone. The researchers concluded that “morphology may not always be a reliable indicator of hybrid status” and that “hybrid zones could comprise a large number of multigenerational backcrossed hybrids that are indistinguishable from the parental species” [55]. These observations are in agreement with our modeling approach, which is based on phenotypes (representing morphology in [55]) but not genotypes (representing genetic characteristics in [55]); this approach is relevant, because natural selection acts on phenotypes and not genotypes. The authors [55] discuss numerous other examples of cryptic hybridization, where molecular methods identified hybrid individuals that could not be distinguished morphologically from the parental species. Importantly for our modeling results, not only were the majority of hybrids phenotypically indistinguishable from the parental species but the remaining minority of hybrids were phenotypically variable, as modeled in the case represented by Figure 8. Thus, our model is relevant not only to asexual but also to conspecific and even heterospecific sexual reproduction.

The model has other limitations, and additional work is needed to more fully understand the nature of degeneracy-driven dynamics in populations. A one-dimensional – considering only a single heritable phenotypic trait – model has been considered here. Realistic degenerate units are multi-dimensional and characterized by numerous traits; these traits may also interact in a pair-wise or more complex fashion. The results of this work are purely theoretical and computational, and will need to be confirmed with experimental and observational evidence. Experimenting with degeneracy may be challenging, as general practical approaches have not yet been elaborated, and quantitative measures of degeneracy are yet to be defined. Various resource distributions, static and dynamic or uniform and non-uniform, need to be assessed for their effects on population changes in order to better approximate real-world resources. Populations with mixed, heritably retained degeneracy ranges (narrow, intermediate, and broad) need to be tested to better approximate real-world populations. As a theoretical exercise and in preparation for the degeneracy-driven technologies of the future [38], it would be interesting to assess the possibility that individual degeneracy ranges vary in time, stochastically or depending on current fitness. In the implementation presented in the current study, trait degeneracy was intrinsic to individuals and had no cost to them, whereas in some realistic populations, broader degeneracy may come at a higher individual or systemic cost, and must be considered as explorative behavior. For example, humoral adaptive immunity invests in diversity and degeneracy of the B cell repertoire through somatic mutagenesis. The effect of the cost of degeneracy on population dynamics needs to be studied. Even with these simplifications, the model shows interesting behavior, offering new insight into the dynamics of degenerate repertoires. Specifically, trait degeneracy is shown to be a driving factor of both static homogeneity and dynamic divergence in populations.

## Conclusions

The results of this model-based study suggest that the two major modes of evolutionary process, dynamic diversification and static homogeneity, may both be driven, in a stable environment, solely by degenerate competition for resource. The results also suggest that the elimination of transitional forms in divergent evolution and the preservation of heritable trait heterogeneity in phenotypic stasis are not driven by adaptive forces but are both the result of the same mechanism stemming from the degeneracy of heritable phenotypic traits. The observed competitive suppression and, ultimately, competitive exclusion define whether a population splits into two or more subgroups or, alternatively, preserves its integrity while becoming more homogenous. The relative dispersion of degeneracy versus the dispersion of resource distribution defines whether the population undergoes diversification or stasis. If the degeneracy of resource consumption is comparable with the range of resource distribution, the population will remain static, and the competitive pressure stemming from degeneracy will increase trait heterogeneity. If the dispersion of degenerate resource consumption is substantially smaller than the range of resource distribution, the population will diversify into several groups, with transitional forms suppressed due to the same degeneracy-driven competitive pressure. As such, static homogeneity and dynamic diversification are two outcomes of the same process, with the difference between them defined by the dispersion of trait degeneracy in a given environment.

## Competing interests

The authors declare that they have no competing interests.

## Authors’ contributions

All authors have made substantial intellectual contributions to this study. NA, MSA, and FA contributed to formulation of the model, analysis and interpretation of the results, and preparation of the manuscript. NA and SPA implemented the model in software. SPA conceived of the study, formulated the model, and analyzed and interpreted the results. All authors read and approved the final manuscript.
